# BRCA2 reversion mutation–independent resistance to PARP inhibition through impaired DNA prereplication complex function

**DOI:** 10.1073/pnas.2426743122

**Published:** 2025-06-03

**Authors:** Kyrie Pappas, Matteo Ferrari, Perianne Smith, Subhiksha Nandakumar, Zahra Khan, Serina B. Young, Justin LaClair, Marco Vincenzo Russo, Emmet Huang-Hobbs, Nikolaus Schultz, Wassim Abida, Wouter Karthaus, Maria Jasin, Charles L. Sawyers

**Affiliations:** ^a^Human Oncology and Pathogenesis Program, Memorial Sloan Kettering Cancer Center, New York, NY 10065; ^b^Developmental Biology Program, Memorial Sloan Kettering Cancer Center, New York, NY 10065; ^c^Department of Microbiology, Immunology, and Cancer Biology, University of Minnesota, Minneapolis, MN 55455; ^d^Computational Oncology Service, Department of Epidemiology and Biostatistics, Memorial Sloan Kettering Cancer Center, New York, NY 10065; ^e^The Halvorsen Center for Computational Oncology, Memorial Sloan Kettering Cancer Center, New York, NY 10065; ^f^Department of Neurosurgery, Columbia University Irving Medical Center, New York, NY 10032; ^g^Albany Medical College, Albany, NY 12208; ^h^Sylvester Comprehensive Cancer Center, Miller School of Medicine, University of Miami, Miami, FL 33136; ^i^Department of Medicine, Memorial Sloan Kettering Cancer Center, New York, NY 10065; ^j^Swiss Institute for Experimental Cancer Research, The École Polytechnique Fédérale de Lausanne, Lausanne, Switzerland 1015; ^k^HHMI, Chevy Chase, MD 20815

**Keywords:** CDT1, homologous recombination, geminin, PARP, prostate

## Abstract

Recent approvals of polymeric adenosine diphosphate ribose (poly(ADP-ribose) polymerase inhibitors (PARPi) for BRCA-mutant metastatic castration resistant prostate cancer necessitate an understanding of the factors that shape sensitivity and resistance. Reversion mutations that restore homologous recombination (HR) repair are detected in ~50 to 80% of BRCA-mutant patients who respond but subsequently relapse, but there is currently little insight into why only ~50% of BRCA-mutant patients display upfront resistance. To address this question, we performed a genome-wide CRISPR screen to identify genomic determinants of PARPi resistance in murine *Brca2^Δ/Δ^* prostate organoids genetically engineered in a manner that precludes the development of reversion mutations. Remarkably, we recovered multiple independent single guide RNAs (sgRNAs) targeting three different members (*Cdt1, Cdc6,* and *Dbf4*) of the DNA prereplication complex (pre-RC), each of which independently conferred resistance to olaparib and the next-generation PARP-1 selective inhibitor AZD5305. Moreover, sensitivity to PARP inhibition was restored in *Brca2^Δ/Δ^*, Cdc6-depleted prostate cells by knockdown of geminin, a negative regulator of Cdt1, further implicating the critical role of a functional pre-RC complex in PARPi sensitivity. Furthermore, ~50% of CRPC tumors have copy number loss of pre-RC complex genes, particularly *CDT1*. Mechanistically, prostate cells with impaired pre-RC activity displayed rapid resolution of olaparib-induced DNA damage as well as protection from replication fork degradation caused by Brca2 loss, providing insight into how Brca2-mutant cancer cells can escape cell death from replication stress induced by PARP inhibition in the absence of HR repair. Of note, a pharmacologic inhibitor that targets the CDT1/geminin complex (AF615) restored sensitivity to AZD5305, providing a potential translational avenue to enhance sensitivity to PARP inhibition.

## Significance

Here, we address a major limitation in the effectiveness of poly(ADP-ribose) polymerase inhibitors (PARPi) in breast cancer gene (BRCA)-mutant prostate cancer treatment: Only ~50% of patients respond despite clear genomic evidence of defective homologous recombination. Prior efforts to study PARPi resistance in prostate cancer have been plagued by the lack of suitable cell lines. We overcame this challenge using primary prostate organoids coupled with genome-wide CRISPR screening. The key finding is that loss-of-function mutations in the DNA prereplication complex (pre-RC) confer PARPi resistance. These genes map to chromosomal regions frequently lost in prostate cancer and could therefore serve as potential biomarkers of treatment response. Pharmacologic inhibition of geminin, a negative regulator of the pre-RC, can restore PARPi sensitivity.

Inhibitors of poly(ADP-ribose) polymerase (PARP) have transformed the treatment of patients with BRCA-mutant breast and ovarian cancers, which typically develop in a background of germline mutations in *BRCA1* or *BRCA2*. More recently, PARP inhibitors (PARPi) have become a critical addition to the treatment of metastatic castration resistant prostate cancer (mCRPC) where, unlike breast and ovarian cancer, ~50% of BRCA mutations occur in the absence of an inherited germline mutation. Furthermore, *BRCA2* mutation/deletion is far more common in prostate cancer than BRCA1 loss of function, whereas in breast cancer, the relative frequencies on *BRCA1* versus *BRCA2* mutation are nearly equal (1). Clinical responses to PARPi are seen in ~50% of BRCA-mutant CRPC patients, as well as in patients with less common alterations that result in HR defects such as *PALB2, FANCA, BRIP1*, and *RAD51B*. Interestingly, response rates in CRPC patients whose tumors have mutations in other DNA damage repair (DDR) pathway genes such as *ATM*, *CDK12,* and *CHEK2* are substantially lower, suggesting that many of these alterations may not result in HR defects of sufficient magnitude to confer synthetic sensitivity to PARP inhibition (2, 3).

Through its PAR activity, PARP-1 modifies thousands of proteins involved in DNA damage response (DDR) and plays a critical role in base excision repair, a key pathway in the repair of single-strand DNA breaks (4). In this context, the cytotoxicity of PARPi in BRCA-mutant cancers is explained by the inability of HR-deficient cells to repair the double-strand DNA breaks that develop following PARP inhibition (5–7). Growing evidence suggests that the cytotoxicity of PARPi in HR deficient cells is not a consequence of blocking PARP enzymatic activity but rather a consequence of trapping PARP on DNA. Trapped PARP1–DNA complexes interfere with DNA replication, and deficiencies in replication fork protection alone (in the setting of intact HR) can result in synthetic lethality to PARPi in specific contexts (8). Indeed, the relative DNA trapping activity of next-generation PARPi is more closely correlated with antitumor activity than with PARP enzyme inhibition (9, 10). Furthermore, genetic deletion or mutation of *PARP1* confers resistance to PARPi therapy in BRCA-mutant cells, indicating that the presence of the protein (perhaps for DNA trapping) rather than enzymatic activity is required for cytotoxic activity (11, 12). These insights, together with the efforts to improve the side effect profile of the first-generation dual PARP-1/2 inhibitor olaparib, have resulted in the development of several PARP-1-selective compounds currently in clinical development (13, 14) (https://ascopubs.org/doi/10.1200/JCO.2024.42.16_suppl.TPS5123).

Despite the clinical success of PARPi across these different cancer settings, both upfront and acquired resistance pose significant limitations to achieving broader clinical benefit. In the case of acquired resistance, reversion mutations in BRCA1 or BRCA2 that restore HR proficiency are found in 50% or more of patients, including prostate cancer (15, 16). Genetic screens in BRCA-mutant cell lines and tumors have identified several mechanisms of reversion-independent resistance such as restoration of HR or replication fork protection, loss of PARP-1 negative regulator PARG, alterations in PARP1, various epigenetic modifications, and P-glycoprotein (PGP)-mediated drug efflux that allow BRCA-mutant cells to survive despite being HR deficient, but the clinical importance of these mechanism are not yet clear (17–22). In prostate cancer, PARPi screens have been performed but not in HR-deficient settings, which poses challenges in knowing whether candidate resistance genes identified in this context will be informative in the clinical context where HR defects are predictive of clinical benefit (23, 24). For example, prior studies in LNCaP sublines with engineered *BRCA2* deletion failed to acquire sensitivity to concentrations of PARPi used clinically (1). PARPi resistance studies in prostate cancer are further challenged by the paucity of PARPi-sensitive BRCA-mutant human prostate cancer cell lines that could be deployed for genetic screens without developing reversion mutations as the primary resistance mechanism (25–28).

To address this challenge, we took advantage of recent developments in prostate organoid technology that allow rapid and efficient generation of complex genotypes that reflect those seen in patients (29, 30). Using this approach, we generated *Brca2^Δ/Δ^* (31) primary mouse prostate organoids in three genetic contexts (*Brca2^Δ/Δ^Trp53^Δ/Δ^*; *Brca2^Δ/Δ^Trp53^Δ/Δ^Pten^Δ/Δ^*; and *Brca2^Δ/Δ^sgRb1*), documented acquisition of exquisite PARPi sensitivity, then performed genome-wide CRISPR screens across all three genotypes to identify sgRNAs enriched in the setting of PARPi treatment. Across all three genetic contexts, we recovered sgRNAs targeting *Cdt1*, *Cdc6,* and *Dbf4* in various combinations, leading us to further investigate the role of the DNA prereplication complex (pre-RC) in response to PARPi therapy. Here, we show that loss-of-function perturbations in the pre-RC complex result in rapid resolution of DNA damage induced by PARP inhibition and rescue from defects in fork protection conferred by *Brca2* loss, ultimately allowing cells to become tolerant to olaparib-induced replication stress. Furthermore, genetic or pharmacologic inhibition of geminin, an upstream suppressor of pre-RC complex activity, restores sensitivity to PARPi in *Brca2^Δ/Δ^* organoids with impaired pre-RC activity, providing a potential translational opportunity.

## Results

### Biallelic *Brca2* Loss in Murine Prostate Organoids Results in Orders of Magnitude Increased Sensitivity to PARP Inhibition.

**Fig. 1.**
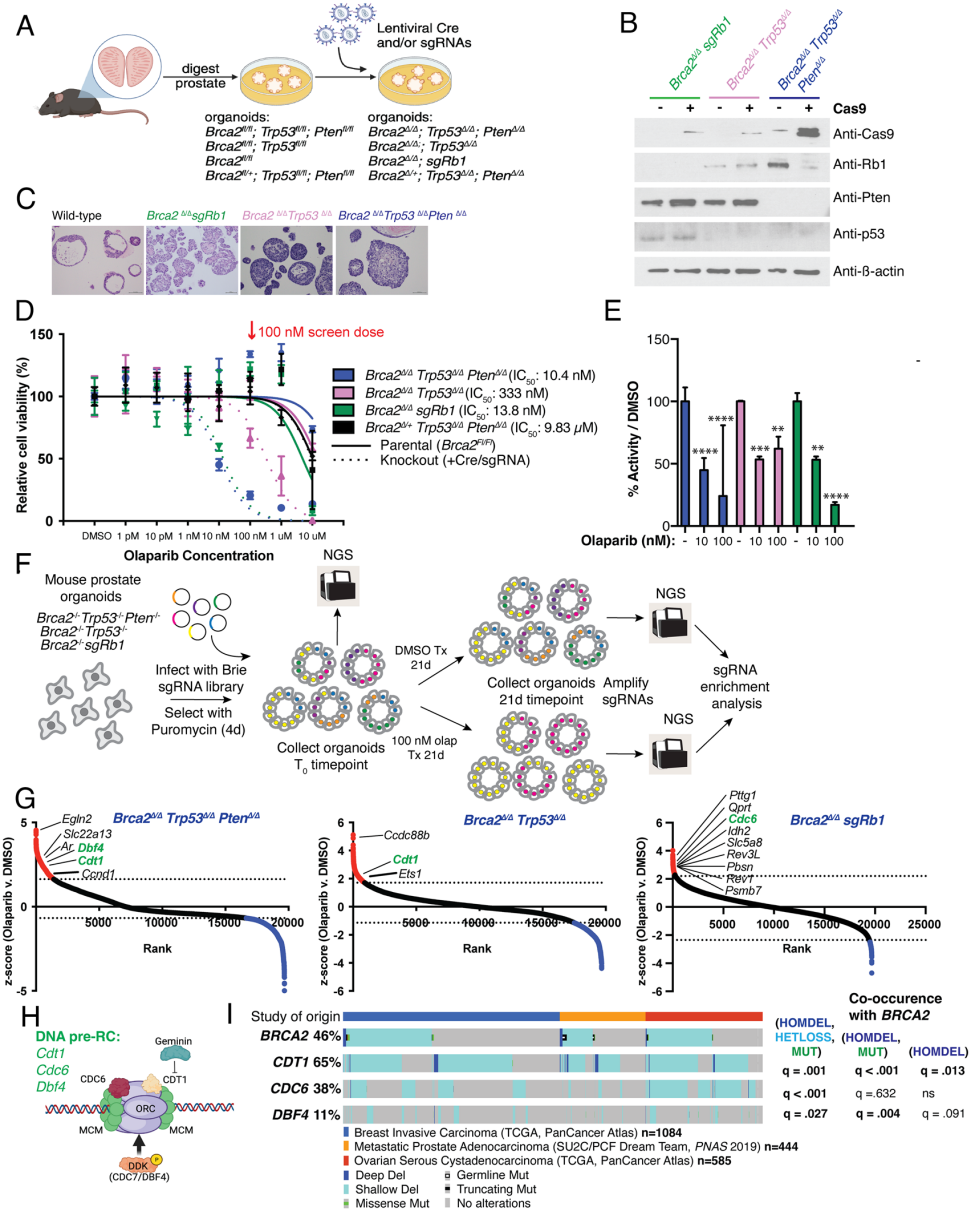
Genome-wide sgRNA screen in Brca2-deficient prostate organoids reveals loss of DNA Pre-RC genes as a putative reversion-independent olaparib resistance mechanism. (*A*) Experimental flow for the generation of Brca2-deficient murine prostate organoid models (created with BioRender.com). Prostates were harvested, and genetic modifications were performed in vitro, as depicted. Created with BioRender.com. (*B*) Immunoblot confirming genotypes (*Rb1, Pten,* and *Trp53*) of Brca2-deficient organoid models (*Brca2^Δ/Δ^ Trp53^Δ/Δ^ Pten^Δ/Δ^, Brca2^Δ/Δ^ Trp53^Δ/Δ^,* and *Brca2^Δ/Δ^ sgRb1)* before and after introduction of stable Cas9 expression prior to screening. β-actin was used as loading control. See *SI Appendix* for *Brca2* genotyping. (*C*) Representative images of H&E stains of *Brca2* wild-type and Brca2-deficient murine prostate organoid lines. (*D*) 7-d olaparib dose–response viability assay in Brca2-deficient murine prostate organoid series, where the genotypes are listed. Viability was assessed using the Incucyte and is displayed as percent inhibition relative to DMSO (vehicle) treatment. Error bars represent the mean ± SD of triplicate technical replicates. 100 nM dose used for screen is indicated (red arrow). Dose–response curves were generated through nonlinear regression analysis. (*E*) PARP1 ELISA-based activity assay was used to quantify PARP-1 activity in Brca2-deficient organoid lines (*Brca2^Δ/Δ^ Trp53^Δ/Δ^ Pten^Δ/Δ^, Brca2^Δ/Δ^ Trp53^Δ/Δ^,* and *Brca2^Δ/Δ^)* following 7-d treatment with dimethyl sulfoxide (DMSO, vehicle) or the indicated doses of olaparib. Data are displayed as % activity relative to DMSO (vehicle). Error bars represent the mean ± SD of triplicate technical replicates. Significance: Two-way ANOVA, Sidak’s correction, ****P < 0.0001, ***P < 0.001, and **P < 0.01. (*F*) Experimental flow of genome-wide sgRNA screen (Brie lentiviral library) for olaparib resistance in Brca2-deficient murine prostate organoid series (*Brca2^Δ/Δ^ Trp53^Δ/Δ^ Pten^Δ/Δ^, Brca2^Δ/Δ^ Trp53^Δ/Δ^*, and *Brca2^Δ/Δ^ sgRb1* lines*).* Collection/sequencing timepoints are indicated (T_0_ is postselection/pre-olaparib treatment, and DMSO /100 nM olaparib samples are collected after 21 d of treatment (~10 cell doublings). (*G*) Rank ordered z-score plots (olaparib versus DMSO treatment) from sgRNA screen for *Brca2^Δ/Δ^ Trp53^Δ/Δ^ Pten^Δ/Δ^, Brca2^Δ/Δ^ Trp53^Δ/Δ^,* and *Brca2^Δ/Δ^ sgRb1* lines (left to right). Each point represents one gene, and the dotted line represents the 5% FDR threshold for enrichment. Pre-RC genes are highlighted in green. Genes that pass the FDR threshold for enrichment are shown in red, and those that pass the threshold for depletion are shown in blue. (*H*) Diagram of Pre-RC complex, showing the screen hits *CDT1, CDC6*, and *DBF4* as subunits, among other proteins (created with BioRender.com). (*I*) Oncoprint (generated by cBioPortal) showing homozygous deletions, shallow deletions, and mutations of *BRCA2, CDT1, CDC6*, and *DBF4*. Studies from breast, ovarian, and prostate cancer are included, and study details are shown. Oncoprint figure legend displays study of origin and the symbols for each type of alteration. Co-occurrence q-values are included for the indicated Pre-RC gene alterations (HOMDEL, HETLOSS, and/or MUT) with *BRCA2*.

In the absence of HR-deficient human prostate cancer cell lines suitable for screening, we chose to model BRCA2 deficiency through Cre-mediated deletion in primary mouse prostate organoids freshly isolated from *Brca2^fl/fl^* mice (Brca2-null, deletion of N-terminal exons 3-4) (32, 33). In contrast to established prostate cancer cell lines, this approach provides the flexibility to model *Brca2* loss in primary cells together with co-occurring genomic alterations commonly seen in BRCA2-mutant human CRPC. Toward that end, we first generated prostate organoid cultures from mice containing a *Brca2^Fl/Fl^* allele alone or in combination with *Trp53^Fl/Fl^*and/or *Pten^Fl/Fl^* alleles. Ex vivo infection with Cre-expressing lentivirus virus was performed to generate organoid lines harboring homozygous loss of *Brca2* and *Trp53* ± *Pten* loss (*Brca2^Δ/Δ^Trp53^Δ/Δ^* and *Brca2^Δ/Δ^Trp53^Δ/Δ^ Pten^Δ/Δ^*). Similarly, we also generated organoids with heterozygous *Brca2* loss (*Brca2*^+/^*^Δ^ Trp53^Δ/Δ^ Pten^Δ/Δ^*) to assess whether haploinsufficiency enhances sensitivity to PARP inhibition. We also modeled combined loss of *BRCA2* and *RB1* based on the fact that recurrent deletions of 13q23, spanning both the *BRCA2* and *RB1* gene loci, are often seen in CRPC (Fig. 1*A*). *Rb1* was targeted in Cre-recombined *Brca2^Fl/Fl^ organoids* using sgRNAs (*Brca2^Δ/Δ^ sgRb1*), followed by ex vivo Cre recombination to delete *Brca2*. Deletion of *Trp53, Pten,* and *Rb1* in the respective organoid cultures was confirmed by western blot (Fig. 1*B*). Recombination of the floxed *Brca2* locus was confirmed by PCR (*SI Appendix*, Fig. S1 *A* and *B*). Histologic analysis across the *Brca2*-deleted organoid series revealed loss of the acinar/luminal/cystic features characteristic of wild-type organoids, likely a consequence of codeletion of *Trp53*, *Pten,* and *Rb1* as these same features are routinely observed following loss of these tumor suppressors in organoids with wild-type *Brca2* (Fig. 1*C*).

Homozygous *Brca2* loss across all three genomic contexts (*Trp53^Δ/Δ^*; *Trp53^Δ/Δ^Pten^Δ/Δ^*; and *sgRb1*) resulted in 30 to 1,000-fold enhanced sensitivity to the PARPi olaparib, depending on the co-occurring genotype (with IC_50_s ranging from 10 to 333 nM, versus 10 μM in parental cells). This is notable because prior attempts to model the synthetic lethality to PARP inhibition seen with BRCA2 loss using human prostate cancer cell lines such as LNCaP yielded modest increases in olaparib sensitivity (low μM) that are not reflective of drug concentrations associated with clinical activity (23, 24). Importantly, monoallelic loss of *Brca2* did not confer sensitivity to olaparib, with an IC_50_ of 10 μM, similar to that of the *Brca2^fl;fl^* lines prior to Cre recombination (Fig. 1*D*). As expected, Parp1 activity (measured by enzyme-linked immunosorbent assay (ELISA)) was inhibited by 50 to 80% at 10 and 100 nM olaparib concentrations in *Brca2*-deficient organoids (Fig. 1*E*). Collectively, these data establish that murine prostate organoid cultures with biallelic loss of *Brca2* accurately mirror the synthetic lethality observed clinically in BRCA-mutant patients treated with PARPi. Furthermore, the large window in IC_50_ between sensitive and resistant genotypes provides an efficient experimental platform to identify genetic determinants of PARPi resistance through large-scale CRISPR screening. Finally, the specific *Brca2^fl/fl^* allele used to generate this model precludes the generation of *Brca2* reversion mutations, allowing us to focus the screen on identifying reversion mutant independent resistance mechanisms.

### Recovery of Multiple Components of the DNA Pre-RC from Genome-Wide CRISPR Screen for PARPi Resistance.

To perform the screen, we introduced a Brie mouse lentiviral library with puromycin selection containing four sgRNAs per gene and 1,000 nontargeting sgRNAs (34) into organoid cultures of three Brca2-deficient genotypes (*Brca2^Δ/Δ^ Trp53^Δ/Δ^*; *Brca2^Δ/Δ^ sgRb1*; and *Brca2^Δ/Δ^ Trp53^Δ/Δ^ Pten^Δ/Δ^*) at a multiplicity of infection of 0.3, and screens were run in duplicate for each organoid line. Cultures were selected for 4 d postinfection, a portion was collected (T_0_), and cells were passaged for 21 d (roughly 10 doublings) in vehicle or 100 nM olaparib, then harvested for sgRNA amplification and next-generation sequencing to identify sgRNAs selectively enriched by olaparib treatment (Fig. 1*F*). Using an FDR enrichment threshold of 5% (experimentally determined for each organoid line based on distribution of the nontargeting sgRNAs) together with a requirement of at least three of four sgRNAs per gene for hit calling (see *Materials and Methods* for details), we generated a list of genes selectively depleted following olaparib treatment in *Brca2^Δ/Δ^ Trp53^Δ/Δ^* organoids (n = 487), *Brca2^Δ/Δ^ sgRb1* organoids (n = 149), and *Brca2^Δ/Δ^ Trp53^Δ/Δ^ Pten^Δ/Δ^* organoids (n = 322) (Fig. 1*G* and *SI Appendix*, Spreadsheet S1).

We then used gene set enrichment analysis to identify pathways represented by these genes, which revealed striking enrichment of multiple gene sets involved in DNA replication and DDR (*SI Appendix*, Fig. S2 and Spreadsheet S2). Through this lens, we then examined specific genes within these pathways and noted enrichment of multiple independent sgRNAs targeting three different members (*Cdt1, Cdc6,* and *Dbf4*) of the DNA pre-RC, a multiprotein complex that controls the spacing and timing of replication fork origin licensing to ensure that DNA is replicated once per cell cycle (depicted in Fig. 1*H*). Furthermore, enrichment of at least one of these three core pre-RC components was seen across all three Brca2-deficient organoid cultures (Fig. 1*G*).

Because a primary goal of the screen was to identify genes whose mutation/deletion might mitigate response to PARPi therapy in CRPC patients, we examined publicly available prostate cancer genomic datasets for copy number loss of *CDT1, CDC6,* and *DBF4* and whether these losses overlap with *BRCA2* mutation status (35, 36). Of these, *CDT1* loss is most common (65% of cases have heterozygous or homozygous loss) and these losses overlap significantly with *BRCA2* loss. *CDC6* and *DBF4* loss are much less common but also show significant overlap with *BRCA2* loss when both heterozygous and homozygous loss are both considered (Fig. 1*I*). Based on these potentially relevant clinical associations, we focused on all three pre-RC genes for deeper investigation.

### Resistance to PARP Inhibition Conferred by Impaired Pre-RC Complex Function Is Rescued by Geminin Knockdown.

**Fig. 2.**
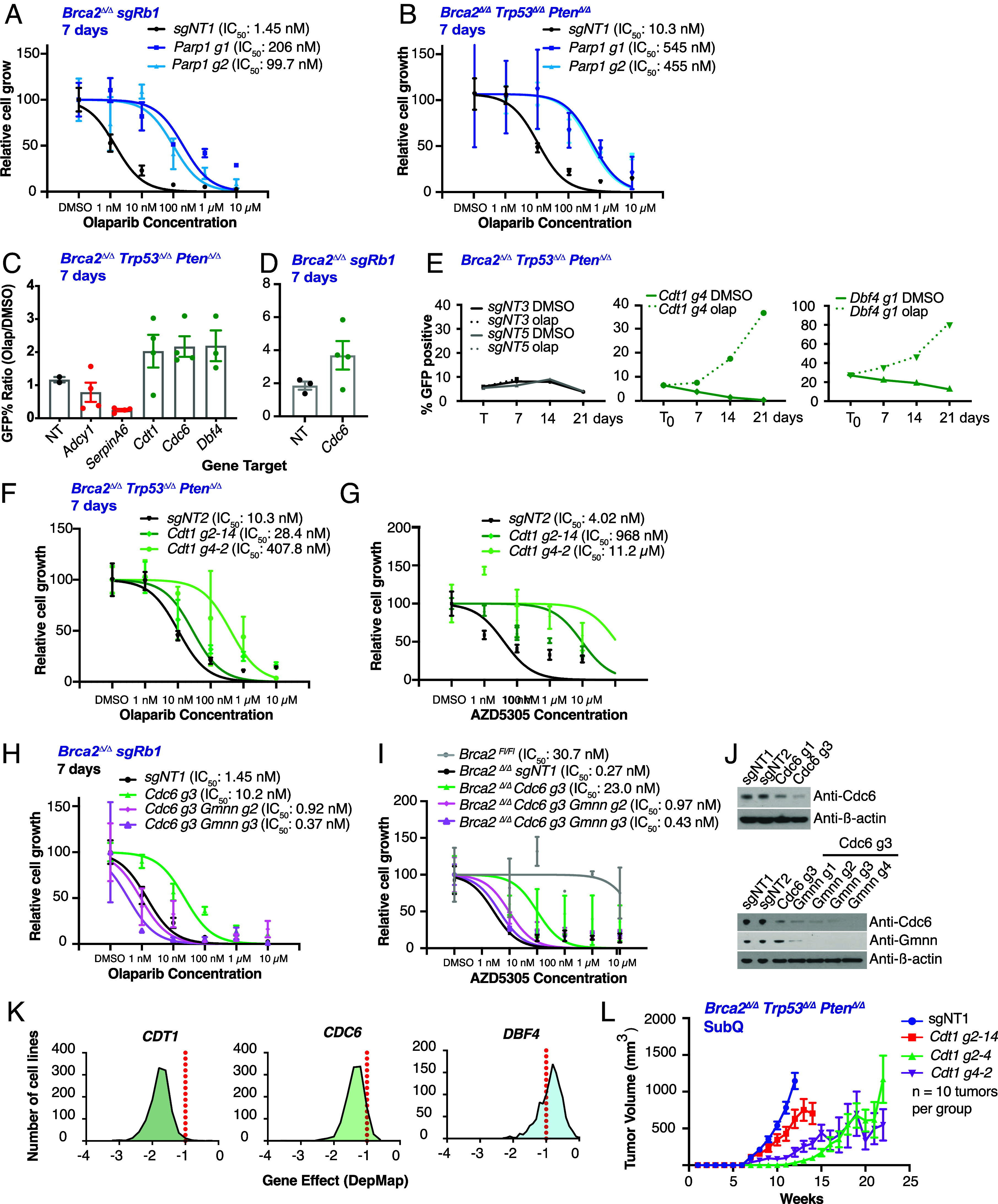
Loss of Pre-RC genes is tolerated and drives PARPi resistance in Brca2-deficient organoid models of mCRPC. 7-d olaparib dose–response viability assay in (*A*) *Brca2^Δ/Δ^ Trp53^Δ/Δ^ Pten^Δ/Δ^,* and (*B*) *Brca2*^Δ/Δ^ *sgRb1* murine prostate organoids stably expressing the indicated sgRNAs (*sgNT1, Parp1 g1,* and *Parp1 g2*). Viability was quantified using the Incucyte and is displayed as percent inhibition relative to DMSO (vehicle) treatment. Error bars represent the mean ± SD of triplicate technical replicates. Dose–response curves were generated through nonlinear regression analysis. (*C* and *D*) GFP competition assays using indicated sgRNAs in LentiCRISPR-GFP treated with 100 nM olaparib or DMSO (vehicle) for 7 d in (*C*) *Brca2^Δ/Δ^ Trp53^Δ/Δ^ Pten^Δ/Δ^*, and (*D*) *Brca2^Δ/Δ^ sgRb1* organoids. Data are displayed as the ratio of %GFP in olaparib/DMSO. Error bars represent the mean ± SEM, and each point is an individual sgRNA. (*E*) Time course GFP competition assays for the indicated sgRNAs after 100 nM olaparib or DMSO (vehicle) treatment for 7, 14, and 21 d in *Brca2^Δ/Δ^ Trp53^Δ/Δ^ Pten^Δ/Δ^* organoids. Data are displayed as % GFP. Olaparib is the dashed line, DMSO (vehicle) is the solid line. GFP was quantified using flow cytometry for (*C*–*E*). (*F* and *G*) 7-d olaparib dose–response viability assay in *Brca2^Δ/Δ^ Trp53^Δ/Δ^ Pten^Δ/Δ^* organoids stably expressing the indicated sgRNAs (*sgNT2, Cdt1 g2-14,* and *Cdt1 g4-2*) in LentiCRISPR-GFP treated with (*F*) olaparib, or (*G*) AZD5305. Viability was quantified using the Incucyte and is displayed as percent inhibition relative to DMSO (vehicle) treatment. (*F* and *G*) Error bars represent the mean ± SD of triplicate technical replicates. Dose–response curves were generated through nonlinear regression analysis. (*H* and *I*) 7-d olaparib dose–response viability assay in *Brca2^Δ/Δ^ sgRb1* organoids stably expressing the indicated sgRNAs (*sgNT1, Cdc6 g3, Gmnn g2,* and *Gmnn g3*) in LentiCRISPR-GFP treated with (*H*) olaparib, or (*I*) AZD5305. Viability was quantified using the Incucyte and is displayed as percent inhibition relative to DMSO(vehicle) treatment. (*H* and *I*) Error bars represent the mean ± SD of triplicate technical replicates. Dose–response curves were generated through nonlinear regression analysis. (*J*) Immunoblot assessing protein levels of Cdc6 and Geminin in *Brca2^Δ/Δ^ sgRb1* organoids stably expressing the indicated sgRNAs (*sgNT1, sgNT2, Cdc6 g1, Cdc6 g3, Gmnn g2,* and *Gmnn g3*) in LentiCRISPR-GFP. β-actin was used as loading control. (*K*) Histograms of aggregate data from the Cancer Dependency Map (DepMap) displaying the effect on viability when expressing sgRNAs targeting *CDT1*, *CDC6*, and *DBF4* (left to right) in 1,178 cell lines. The gene effect value of −1 is the standard cutoff for essential genes and is indicated by the dashed red line. (*L*) *Brca2^Δ/Δ^ Trp53^Δ/Δ^ Pten^Δ/Δ^* organoids expressing the indicated sgRNAs (*sgNT1, Cdt1 g2-14, Cdt1 g2-4,* and *Cdt1 g4-2*) in LentiCRISPR-GFP were injected into NSG mice, tumor volume was measured weekly, displayed in mm^3^. Error bars represent mean ± SEM, n = 10 tumors per group.

Loss of *PARP1* has been reported to promote PARPi resistance in multiple contexts, and *Parp1* sgRNAs were recovered from the screen in *Brca2^Δ/Δ^ Trp53^Δ/Δ^* organoids, likely implicating the DNA trapping mechanism for PARPi-induced synthetic lethality as opposed to inhibition of enzymatic PARylation activity (11). To validate PARP-1 as a screen hit, we expressed two independent sgRNAs against *Parp1* in *Brca2^Δ/Δ^ sgRb1* and *Brca2^Δ/Δ^ Trp53^Δ/Δ^ Pten^Δ/Δ^* organoids (Fig. 2 *A* and *B*). Organoids tolerated PARP-1 knockout and were resistant to olaparib, presumably due to loss of DNA trapping.

To confirm the robustness of our sgRNA hit calling and validate the role of pre-RC complex in PARPi sensitivity, we used green fluorescent protein (GFP) competition assays to compare the relative enrichment of all pre-RC complex sgRNA hits versus various controls (nontargeting sgRNAs as well as nonenriched sgRNAs) in *Brca2^Δ/Δ^ Trp53^Δ/Δ^ Pten^Δ/Δ^* and *Brca2^Δ/Δ^sgRb1* organoids (see *Materials and Methods* for details on enrichment screen). sgRNAs targeting pre-RC complex members *Cdt1, Cdc6,* and *Dbf4* were enriched in 100 nM olaparib treatment versus DMSO (vehicle), whereas sgRNAs targeting *Adcy1* or *SerpinA6* (not enriched in the screen) and nontargeting sgRNAs (*sgNT3* and *sgNT5*) were not (Fig. 2 *C*–*E*).

With the growing recognition that PARP-1 is likely the critical target for PARPi-induced synthetic lethality in HR defective cancers, there is intense interest in developing compounds with improved PARP1 selectivity. AZD5305 (saruparib) is one such compound currently in phase 3 clinical development in CRPC (13, 14) (https://ascopubs.org/doi/10.1200/JCO.2024.42.16_suppl.TPS5123). Side-by-side viability assays comparing olaparib and AZD5303 confirmed multilog enhanced sensitivity in *Brca2^Δ/Δ^ Trp53^Δ/Δ^ Pten^Δ/Δ^* organoids as well as rescue by sgRNA targeting of *Cdt1*, a core component of the pre-RC (Fig. 2 *F* and *G* and *SI Appendix*, Fig. S3).

The pre-RC complex is tightly regulated, most notably by geminin which sequesters Cdt1 through physical interaction when origin licensing is not needed. Having validated loss of pre-RC complex as a cause of PARPi resistance, we next asked whether we could restore PARPi sensitivity through geminin knockdown. Because the sequestering effect of geminin is specific to Cdt1, we first established PARPi resistance through introduction of sgRNAs targeting *Cdc6* in *Brca2^Δ/Δ^ sgRb1* organoids, then similarly targeted geminin to release any sequestered Cdt1 with the goal of potentially restoring pre-RC activity. Remarkably, combined deletion of *Cdc6* and geminin restored sensitivity to olaparib and to AZD5305 to levels comparable to parental cells (Fig. 2 *H*–*J*). Collectively, these results confirm the importance of the pre-RC complex as a determinant of PARPi sensitivity.

It is worth noting that *CDT1*, *CDC6,* and *DBF4* are all classified as essential genes in yeast and human in the most recent update of the DEG database (37). To better understand how BRCA2-mutant prostate organoids can tolerate deletion of these pre-RC complex genes, we examined the dependency profiles of *CDT1, CDC6,* and *DBF4* in the cancer cell line dependency map (DepMap; https://depmap.org/portal/). Using a gene effect score of <−1 as a threshold for dependency, *CDT1* loss was not tolerated across most cancer cell lines whereas the consequences of *CDC6* and *DBF4* loss were less broad across the panel (Fig. 2*K*). To investigate the consequences of *Cdt1* loss in our prostate model, we evaluated the in vivo tumorigenicity of *Brca2^Δ/Δ^ Trp53^Δ/Δ^ Pten^Δ/Δ^* organoids with and without *Cdt1* deletion. Three independent clonal *Cdt1*-edited sublines all gave rise to tumors, albeit with slower growth rates than parental Cdt1-intact organoids (Fig. 2*L*). Importantly, complete *Cdt1* deletion was confirmed by isolating clones and sequencing across the sgRNA-edited locus in all three organoid sublines prior to injection (clones characterized in *SI Appendix*, Fig. S3 *A* and *B*. The resulting tumors also retained a high fraction of *Cdt1* edited alleles (*SI Appendix*, Figs. S4–S6), indicating that *Cdt1* loss is tolerated in this genomic context.

### PARPi-Induced DNA Damage Is Resolved and Stalled Replication Forks Recover in *Brca2^Δ/Δ^* Cells with Impaired Pre-RC Function.

**Fig. 3.**
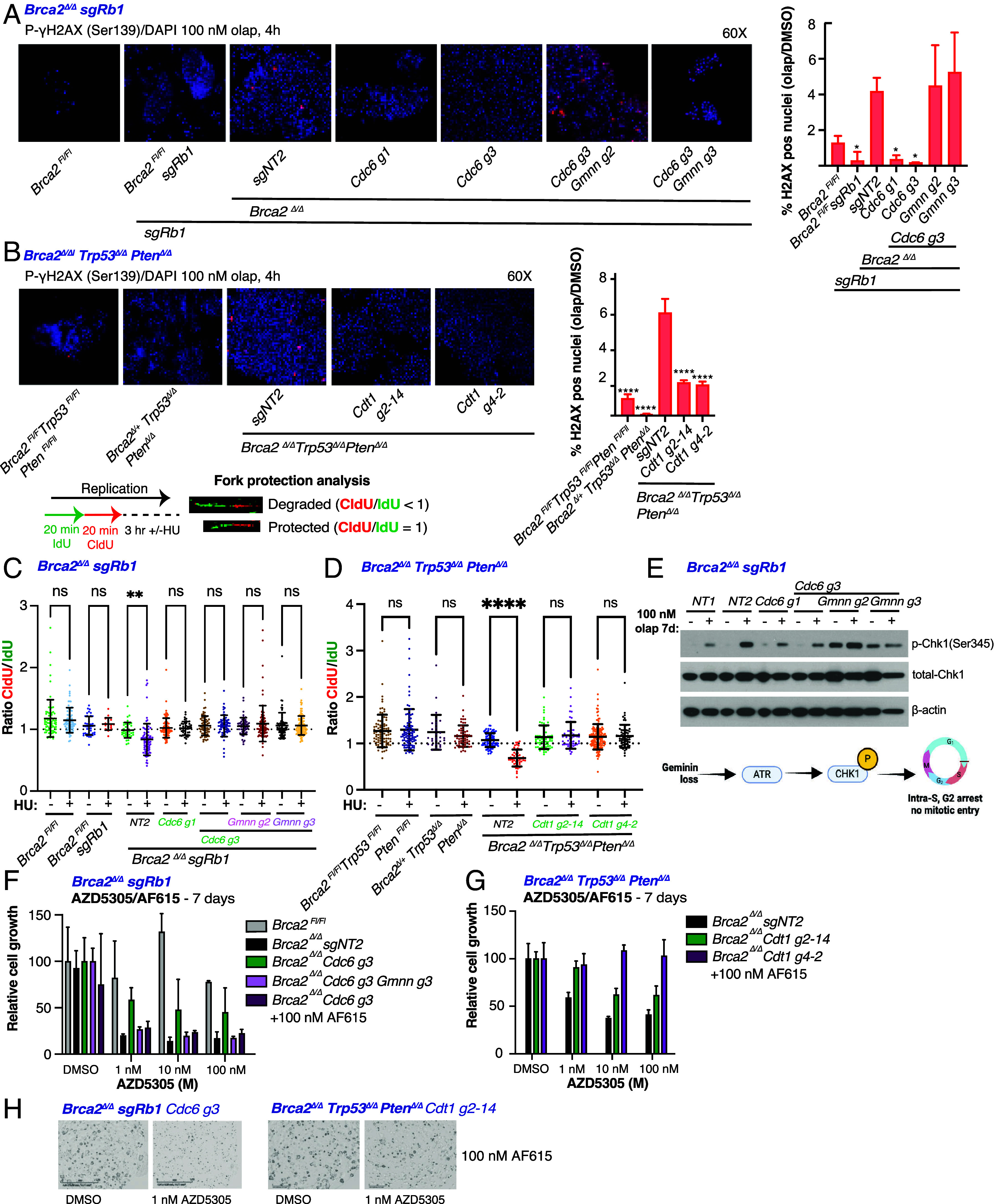
Loss of Pre-RC genes leads to PARPi resistance through the resolution of double-strand breaks and rescued fork protection and can potentially be exploited by targeted therapy. (*A* and *B*) Representative 60× composite immunofluorescence images for γ-H2AX (red) and DAPI (blue) following 100 nM olaparib treatment for 4 h, quantifications shown on *Right*. (*A*) *Brca2^Δ/Δ^ sgRb1* organoids stably expressing the indicated sgRNAs (*sgNT1, sgNT2, Cdc6 g1, Cdc6 g3, Gmnn g2,* and *Gmnn g3*) in LentiCRISPR-GFP. *Brca2^Fl/Fl^* and *Brca2^Fl/Fl^sgRb1* organoids were included as controls. (*B*) *Brca2^Δ/Δ^ Trp53^Δ/Δ^ Pten^Δ/Δ^* organoids expressing the indicated sgRNAs (*sgNT1, Cdt1 g2-14,* and *Cdt1 g4-2*) in LentiCRISPR-GFP. *Brca2^Fl/Fl^ Trp53^Fl/Fl^ Pten^Fl/Fl,^* and *Brca2^Δ/+^ Trp53^Δ/Δ^ Pten^Δ/Δ^* organoids were included as controls. Quantifications (*A* and *B*) are the ratio of % positive γ-H2AX in olaparib/DMSO treated organoids. Error bars represent the mean ± SD of triplicate technical replicates, and significance was calculated by one-way ANOVA relative to the nontargeting (sensitive) organoid line. (*C* and *D*) DNA fiber analysis was performed where cells were treated with IdU (green) and CldU (red) for 20 min each, followed by hydroxyurea (HU) for 3 h. Data are displayed as a ratio of IdU/CldU track length, where a ratio <1 indicates a degraded fork, and a ratio of one indicates a protected fork. (*C*) DNA fiber analysis in *Brca2^Δ/Δ^ sgRb1* organoids stably expressing the indicated sgRNAs (*sgNT1, sgNT2, Cdc6 g1, Cdc6 g3, Gmnn g2,* and *Gmnn g3*) in LentiCRISPR-GFP. *Brca2^Fl/Fl^* and *Brca2^Fl/Fl^sgRb1* organoids were included as controls. (*D*) DNA fiber analysis in *Brca2^Δ/Δ^ Trp53^Δ/Δ^ Pten^Δ/Δ^* organoids expressing the indicated sgRNAs (*sgNT1, Cdt1 g2-14,* and *Cdt1 g4-2*) in LentiCRISPR-GFP. *Brca2^Fl/Fl^ Trp53^Fl/Fl^ Pten^Fl/Fl,^* and *Brca2^Δ/+^ Trp53^Δ/Δ^ Pten^Δ/Δ^* organoids were included as controls. (*E*) Immunoblot assessing protein levels of p-Chk1(Ser345) and total Chk1 in *Brca2^Δ/Δ^ sgRb1* organoids expressing the indicated sgRNAs (*sgNT1, sgNT2, Cdc6 g1, Cdc6 g3, Gmnn g2,* and *Gmnn g3*) in LentiCRISPR-GFP following DMSO (vehicle) or 100 nM olaparib treatment for 7 d. β-actin was used as loading control. (*F*) Viability assay in *Brca2^Δ/Δ^ sgRb1* organoids expressing the indicated sgRNAs (sgNT2, Cdc6 g3, and Gmnn g3) in LentiCRISPR-GFP following treatment with DMSO (vehicle) or the indicated doses of AZD5305 and AF615 for 7 d. *Brca2^Fl/Fl^* organoids were included as controls. (*G*) Viability assay in *Brca2^Δ/Δ^ Trp53^Δ/Δ^ Pten^Δ/Δ^* organoids expressing the indicated sgRNAs (*sgNT1* and *Cdt1 g2-14*) in LentiCRISPR-GFP following treatment with DMSO (vehicle) or the indicated doses of AZD5305 and AF615 for 7 d. *Brca2^Fl/Fl^ Trp53^Fl/Fl^* were included as controls. Viability assay was quantified using the Incucyte and is displayed as percent inhibition relative to DMSO (vehicle) treatment. Error bars represent the mean ± SD of triplicate technical replicates. (*H*) Representative photos for indicated organoid lines following AZD5305/AF615 combination treatment. Photos were captured by the Incucyte. ****P < 0.0001, **P < 0.01, and *P < 0.05.

PARPi induce DNA damage. In HR-competent cells, this damage is repaired, but in cells lacking HR, dsDNA breaks accumulate resulting in cytotoxicity (7). To determine whether PARPi-induced DNA damage in *Brca2^Δ/Δ^* prostate organoids with pre-RC complex dysfunction persists or is resolved, we measured γH2AX foci, a widely used marker of DNA damage, after 4 h of olaparib treatment. As expected, γH2AX foci were induced in *Brca2^Δ/Δ^ sgRb1* and *Brca2^Δ/Δ^ Trp53^Δ/Δ^ Pten^Δ/Δ^* organoids, indicative of the accumulation of DSBs (Fig. 3 *A* and *B*). In *Brca2^Δ/Δ^ sgRb1* organoids lacking Cdc6, these foci were resolved by 4 h but not in those with concurrent geminin knockdown (Fig. 3*A*). Similarly, γH2AX foci were resolved at 4 h in *Brca2^Δ/Δ^ Trp53^Δ/Δ^ Pten^Δ/Δ^* organoids with Cdt1 knockdown (Fig. 3*B*). Taken together, these results establish that reversion mutation-independent resistance to PARPi conferred by loss of pre-RC complex mutation is associated with resolution rather than tolerance of PARPi-induced DNA damage.

In addition to its role in HR, one of the central functions of Brca2 in DNA replication is to protect stalled replication forks from collapse. Since pre-RC complex deficient *Brca2^Δ/Δ^* organoids can overcome the genotoxic stress of PARP inhibition in the absence of HR repair, we asked whether fork protection was restored in resistance phenotypes driven by loss of pre-RC activity. We used DNA fiber analysis to visualize individual replication forks under hydroxyurea (HU)-mediated replication stress (Fig. 3*C*). *Brca2^Δ/Δ^sgRb1* murine prostate organoids displayed the expected fork protection defect caused by Brca2 deficiency (CldU/IdU ratio < 1), which was not present in *Brca2^Fl/Fl^ or Brca2^Fl/Fl^sgRb1* organoids (Fig. 3*C*). Upon introduction of sgRNAs against Cdc6, the fork protection defect was fully rescued (Fig. 3*C*). Similarly, *Brca2^Δ/Δ^ Trp53^Δ/Δ^ Pten^Δ/Δ^* organoids displayed an identical fork protection defect, which was rescued by sgRNAs against *Cdt1* (Fig. 3*D*). As expected, fork speed was not affected by loss of Pre-RC members (*SI Appendix*, Fig. S7 *A* and *B*).

Interestingly, although the addition of *Gmnn* sgRNAs concurrently with *Cdc6* loss restored PARPi sensitivity, the fork protection defect was not similarly rescued (Fig. 3*C*), suggesting that Geminin loss may promote sensitivity through an alternative mechanism. These results demonstrate that loss of pre-RC members can rescue the fork protection defect conferred by *Brca2* loss and confer PARPi resistance. The finding that Geminin loss does not restore the fork protection defect yet confers PARPi sensitivity likely reflects the distinct roles of Cdc6 in origin licensing during G1, and Gmnn in regulating/sequestering Cdt1 during S and G2. Geminin loss has the potential to be more detrimental, resulting in origin licensing by Cdt1 at inappropriate times in the cell cycle, leading to mitotic catastrophe.

To further investigate how *Brca2^Δ/Δ^* organoids respond to the DNA damage induced by PARPi, we interrogated the ATR/Chk1 checkpoint and found that *Brca2^Δ/Δ^sgRb1* organoids activate p-Chk1 in response to olaparib treatment in cells expressing control sgRNAs (*NT1* and *NT2*) and Cdc6 (Fig. 3 *E*, *Left*). Of note, Gmnn knockdown on top of Cdc6 knockout resulted in constitutive Chk1 phosphorylation at baseline, in the absence of PARPi treatment (Fig. 3*E*), which increased further following olaparib exposure. This result is consistent with previous work showing that Geminin loss results in increased baseline activation of the intra-S checkpoint due to overreplication, ultimately leading to G2-arrest and mitotic catastrophe (38, 39). Whereas pre-RC dysfunction is sufficient to confer PARPi resistance in the absence of HR (in part through rescue of stalled replication forks), we postulate that geminin loss causes an increase in replication stress above the threshold for viability.

A recent report describing a small molecule inhibitor of the Geminin–CDT1 interaction (AF615) (40) prompted us to investigate the activity of this compound in *Brca2^Δ/Δ^ sgRb1 Cdc6 g3* organoids treated with escalating concentrations of AZD5305 (Fig. 3 *F* and *H*). Resistance to AZD5305 driven by loss of Cdc6 was completely reversed by AF615, even at 1 nM AZD5305. Conversely, AF615 did not restore AZD5305 sensitivity to *Brca2^Δ/Δ^ Trp53^Δ/Δ^ Pten^Δ/Δ^ sgCdt1* organoids, an expected result because the sequestering effect of geminin requires Cdt1 (Fig. 3 *G* and *H*). These results suggest a potential translational opportunity to explore the use of Geminin inhibitors to restore PARPi sensitivity in BRCA2-deficient cancers in certain contexts.

## Discussion

The treatment landscape of BRCA-mutant cancers has been revolutionized by PARPi therapy and continues to improve with the development of more potent, PARP1-selective compounds that enhance tolerability and should facilitate exploration of combination therapies. Nonetheless, PARPi resistance is a significant challenge that limits long-term clinical benefit. In patients who relapse after an initial response to therapy, reversion mutations in the *BRCA* locus that restore HR repair are, by far, the most frequent cause of acquired resistance (15). However, our understanding of upfront resistance (i.e., failure to respond to initial treatment) in patients is less mature despite multiple efforts to define genetic determinants of PARPi sensitivity and resistance through CRISPR screens using experimental models.

Here, we have approached this question through the lens of *BRCA*-mutant prostate cancer which, in contrast to breast and ovarian cancer, is largely a *BRCA2*-mutant disease with a higher percentage of somatic rather than germline cases, as well as frequent 13q copy number loss spanning the *BRCA2* gene. Prior PARPi screens in prostate cancer have been limited by a paucity of available BRCA-mutant human prostate cancer cell lines, as well as by the inability to accurately replicate the synthetic lethality phenotype of PARPi treatment using isogenic *BRCA*-wild-type/*BRCA*-mutant cell line models. Our approach differs in that we used primary mouse prostate organoid cultures to generate *Brca2*-mutant genotypes that, gratifyingly, mirror those seen in patients and acquire exquisite PARPi sensitivity (orders of magnitude reduction in IC_50_, low nanomolar range for olaparib). Furthermore, the genetic approach used to delete *Brca2* precludes the development of reversion mutations, allowing us to focus the screen on reversion-independent PARPi resistance mechanisms.

Among many validated hits, we were struck by the recovery of multiple independent sgRNAs to three core members of the DNA pre-RC (*Cdt1, Cdc6,* and *Dbf4*) across all three of the genetic contexts used in the screen. We show that loss of these individual pre-RC members promotes resistance through resolution rather than tolerance of DNA damage and through rescued fork protection, allowing S-phase completion and cell cycle progression. Further efforts are needed to elucidate possible alternative DNA repair pathways facilitating the resolution of DNA damage following PARPi treatment in cells with impaired Pre-RC function and whether trapping of Parp1 by PARPi influences these resistance phenotypes. In addition to γH2AX, measuring 53BP1 and RPA foci formation in resistant cells could provide more resolution on the type of DNA damage being resolved. Depletion of fork reversal enzymes has been attributed to increased fork protection, a finding that warrants further investigation in the context of PARPi resistance conferred by pre-RC loss. The finding that enhancing pre-RC activity through *Gmnn* knockout in Cdc6-deficient organoids restores PARPi sensitivity not only validates that loss of pre-RC function confers PARPi resistance but also provides a potential path to address this challenge clinically through small molecule disruption of the Geminin–CDT1 complex (40). The Geminin knockout experiments also provide insight into the mechanism of restored PARPi sensitivity, likely through the higher level of baseline replication stress (as measured by elevated pChk1) that can no longer be tolerated following PARPi exposure.

The fact that genomic loss of several pre-RC complex proteins (CDT1, CDC6, and DBF4) is evident in CRPC patients raises the possibility that these alterations could be genomic determinants of upfront PARPi response. Our preliminary efforts to address this possibility, while intriguing, are limited by small cohorts of genomically annotated patients with longitudinal clinical response data. As larger cohorts of CRPC patients treated with PARPi accrue, it will be important to specifically annotate copy number and mutation status of pre-RC complex genes as these are not routinely included in most cancer gene sequencing panels. Another important question is whether the pre-RC findings reported here in the BRCA2-mutant setting extend to cancers with BRCA1 loss, and whether the genetic background, such as loss of p53, PTEN, and/or RB1, influences the resistance phenotype. Loss of *Cdt1* promoted resistance in both of the p53-deficient organoid lines tested in our study, though larger data cohorts are needed to fully understand the complex relationships between these resistance mechanisms and genetic background. It is also worth considering whether loss-of-function alterations in the pre-RC could be a more generalizable mechanism of resistance to other chemotherapies that induce replication stress such as topoisomerase inhibitors. Further efforts are also warranted to determine whether these resistance mechanisms are relevant to genetic backgrounds other than BRCA1/2 loss that result in HR and/or replication fork protection deficiencies.

## Materials and Methods

### Establishment of Organoid Cell Lines.

Prostates were harvested from a series of *Brca2*-floxed mouse strains: *Brca2^fl/fl^*, *Brca2^fl/fl^ Trp53^fl/fl^*, and *Brca^fl/fl^ Trp53^fl/fl^ Pten^fl/fl^*. The *Brca2^Fl/Fl^* allele used was previously published and does not produce functional protein (Brca2-null) (31). Prostates were minced, digested with 5 mg/mL collagenase type II followed by TrypLE (ThermoFisher, 12605010), and cultured according to published protocols (34, 41). These cultures were used to establish the Brca2-deficient organoid models for this study, and additional detail is provided below.

### Organoid Cell Culture.

Organoid lines were cultured at 37 °C, 5% CO_2_ in Matrigel (Corning, 356231), where 35 μL domes of Matrigel containing the cells/organoids are placed on standard untreated plates for nonadherent cells (any brand/size), allowed to solidify for 20 min, and medium is placed on top to fully submerge the Matrigel discs. Mouse prostate organoids were grown in standard mouse prostate organoid medium composed of 1× Advanced Dulbecco’s Modified Eagle’s Medium (DMEM)/F-12 (Thermo Fisher Scientific, 12634028), 10% Noggin conditioned medium, 5% R-Spondin conditioned medium, 1% Pen/strep (Fisher Scientific, 15-140-122), 1% Glutamax (Thermo Fisher Scientific, 35050079), 10 mM 4-(2-hydroxyethyl)-1-piperazineethanesulfonic acid (HEPES) (pH 7.4), 1× B27 (Fisher Scientific, 17504-044), 200 nM A83-01 (Tocris, 2939), and 1.25 mM N-acetylcysteine (Sigma-Aldrich, A9165). Medium was supplemented with 5 to 50 ng/mL EGF (PeproTech, 315-09), 20 μM Y-27632 (Selleck Chemicals, S1049), and 1 nM DHT (Sigma Aldrich, D-073). Cells were split every 2 to 3 d, mechanically disrupting the cells using fire-polished pipettes each time. All cell lines were confirmed to be free of *Mycoplasma* through regular testing using the Lonza kit (LT07-418).

### Production of Virus.

Lentiviruses were produced by cotransfection of 293 T cells (Takara, 632180) using Lipofectamine 2000 (Invitrogen, 11668) with lentiviral backbone constructs and packaging vectors (psPAX2 and pMD2.G; Addgene, 12260 and 12259). After 2 to 3 d, medium containing virus was collected, passed through a 0.45 μM filter (EMD Millipore, SE1M179M6), and concentrated (System Biosciences, LV825A-1). Virus was resuspended in organoid medium containing 8 μg/mL of polybrene (EMD Millipore, TR-1003-G) and stored at −80 °C (if not used immediately after harvest).

### Genetic Modification of Organoid Lines.

For lentiviral infections, established organoid lines were trypsinized (ThermoFisher, 12605010) for 5 to 10 min and placed in standard nonadherent cell culture plates (without Matrigel) in organoid medium. The minimum amount of medium needed to cover the bottom of the well was used. 8 μg/mL of polybrene (EMD Millipore, TR-1003-G) was added to the infection medium to improve infection efficiency. A spinfection was performed at 500× g for 1 h, and the cells were then placed in a 37 °C, 5% CO_2_ incubator overnight. Cells were collected from the plate, trypsinized briefly if any attachment has occurred, and replated in 3-Dimensional (3D) culture (Matrigel, normal conditions described above). Organoids were allowed to establish for 24 h prior to starting antibiotic selection. Lentiviral Cre was delivered in vitro to recombine floxed tumor suppressor genes (*Brca2, Trp53,* and *Pten*), and recombined cells were selected with 1 µg/mL puromycin (InvivoGen, ant-pr-1).

CRISPR/Cas9 targeting of desired genes was achieved using sgRNAs in LentiCRISPRv2 containing various selection markers. Cells expressing sgRNAs targeting *Rb1* were selected with 10 µg/mL Blasticidin (InvivoGen, ant-bl-1; Addgene, 98290). Postscreen, cells expressing sgRNAs against putative resistance drivers in LentiCRISPRv2GFP (Addgene, 82416) were isolated by sorting for GFP expression 2 to 3 d after infection. These sgRNAs were validated sequences from the mouse Brie library (Addgene, 52963). sgRNAs were cloned into the appropriate LentiCRISPRv2 vector using established protocols (42).

### Organoid Dosage Response Assays.

Cells of interest were seeded in triplicate at 3,000 to 3,500 cells per Matrigel dome per well using a 48-well non-TC-treated plate (Greiner Bio-One, 677102). Drug treatments were prepared in standard mouse prostate organoid medium supplemented with 5 ng/mL EGF (PeproTech, 315-09), 20 uM Y-27632 (Selleck Chemicals, S1049), and 1 nM DHT (Sigma Aldrich. D-073) at concentrations of 1 nM, 10 nM, 100 nM, 1 μM, and 10 μM. Dimethyl sulfoxide (DMSO) (Fisher Scientific, BP231-100) was used as vehicle control. After receiving medium containing drug, cells were placed in the Incucyte S3 which was programmed to take one photo per well every 6 h. % inhibition (relative to vehicle DMSO) was calculated using Microsoft Excel, and dose–response curves (generated by nonlinear regression analysis) were performed using Prism 10, after 7 d of treatment.

### Positive Selection CRISPR Screen.

Cas9 was stably expressed in the LentiCRISPR-Hygro vector, and expression was confirmed by western bot. Doubling time for each organoid line was measured by manual counting at defined timepoints and was ~48 h for Brca2-deficient mouse prostate organoid lines (*Brca2^Δ/Δ^ Trp53^Δ/Δ^*; *Brca2^Δ/Δ^ Rb1^Δ/Δ^*; and *Brca2^Δ/Δ^ Trp53^Δ/Δ^ Pten^Δ/Δ^*). The Brie lentiviral library was prepared by the Gene Editing and Screening core at MSKCC (Addgene, #52963).

The pooled mouse Brie library consisted of four sgRNAs per gene for 19,674 genes in the mouse genome along with 1,000 nontargeting sgRNAs, where each guide is expressed in the LentiGuide-Puro vector. Brca2-deficient mouse prostate organoids were infected with the Brie sgRNA library at a multiplicity of infection of 0.3 and placed under puromycin selection for 4 d. Cells were then either harvested (timepoint T_0_) or placed under DMSO (Fisher Scientific, BP231-100) or 100 nM olaparib (LC Laboratories) treatment for 21 d (~10 doublings). After 21 d, cells were collected, pelleted, and resuspended in 1× phosphate-buffered saline (PBS) and submitted to the Gene Editing & Screening core at MSKCC where libraries were prepared, and the samples were sent for sequencing at the Integrated Genomics Operation at MSKCC. Screens were run in duplicate for each organoid line. Data were analyzed by the Gene Editing and Screening Core (read counts) and the Bioinformatics Core (z-scores and FDR thresholds), both at MSKCC. The guides used to individually test putative gene drivers of resistance (identified from screen) were validated sgRNA sequences from the mouse Brie library and were cloned into the LentiCRISPR-GFP (Addgene, #82416) vector to enable GFP quantification and sorting (instead of antibiotic selection).

### Immunoblotting.

Cells of interest were lysed in 1× radioimmunoprecipitation assay (RIPA) buffer (EMD Millipore, 20-188) supplemented with phosphatase (Fisher, 52-462-51SET) and protease inhibitors (Sigma-Aldrich, 11697498001). Protein content was quantified using Pierce BCA Assay kit (Pierce, A65453) and lysates were prepared in Laemmli sample buffer (BioRad Laboratories, 1610747) supplemented with 10% 2-mercaptoethanol (BioRad Laboratories, 1610710). Samples were resolved using 4 to 12% Bis-Tris gel (Thermo Scientific, NP0335BOX) and blotted onto polyvinylidene fluoride (PVDF) membrane (EMD Millipore, IPVH00010) by wet transfer. Membranes were blocked with 5% milk in tris-buffered saline with Tween20 (TBST) for 1 h before being incubated with primary antibody in 5% milk-TBST at 4 °C overnight. Membranes were then rinsed in TBST and incubated with secondary antibodies in 5% milk-TBST for 1 h. Membranes were visualized using the ECL Prime kit (Cytiva, RPN2232) and autoradiography film (Genesee Scientific, 30-101L).

### GFP Competition Assay.

*Brca2^Δ/Δ^* GFP^+^ sgRNA-expressing cells (previously isolated by flow cytometry) were mixed with unlabeled parental cells at a ratio of 15 to 85%. These cells were plated at 2,500 cells per Matrigel dome and 7,500 cells per well (three domes) in a 12-well plate. For each sgRNA tested, two wells for DMSO treatment and two wells for 100 nM olaparib treatment were plated. The proportion of GFP^+^ cells was measured every 7 d for up to 21 d.

### Immunofluorescence.

Immunofluorescence for γH2AX (Abcam, ab11174) and DAPI was performed by the Molecular Cytology Core Facility (MSKCC) using established protocols and imaged on a Nikon Ti2 Eclipse microscope with a 60× oil immersion objective. γH2AX positive cells and total cells in each image were manually counted on Fiji software.

### PARP Activity Assay.

Brca2-deficient organoids were treated with DMSO (vehicle) (Fisher Scientific, BP231-100), 10 nM olaparib, or 100 nM olaparib (LC Laboratories) for 7 d. Used PARP Pharmacodynamic ELISA Kit II (R&D Systems, 4520B-096-K) according to the manufacturers protocol for suspension cells to quantify PARP1 activity. Results were quantified on a SpectraMax M Series Multi-Mode Microplate Reader.

### DNA Fiber Analysis.

This protocol is based on methods shared by Maria Jasin’s laboratory (MSKCC) for 2D cells and adapted for organoids grown in 3D. Mouse prostate organoid lines (*Brca2^Δ/Δ^ Rb1^Δ/Δ^*; *Brca2^Δ/Δ^ Trp53^Δ/Δ^*; and *Brca2*^Δ/Δ^ *Trp53*^Δ/Δ^ *Pten*^Δ/Δ^) were seeded at 3,000 cells per Matrigel dome and allowed to expand for 1 wk. 10 to 20 domes are needed per condition. Organoids were dissociated with a fire-polished pipette, spun down, and resuspended in fully supplemented organoid medium containing 200 µM IdU and incubated in a 37 °C shaker for 20 min, covered from light. Cells were washed 3× with 1× PBS and resuspended in fully supplemented organoid medium containing 400 µM CldU (Sigma, C6891-100MG) for 20 min in a 37 °C shaker, covered from light. Cells were washed 3× with 1× PBS and resuspended in fully supplemented organoid medium containing DMSO or 100 µM hydroxyurea. Cells were incubated for 3 h on a 37 °C shaker, covered from light. Cells were washed with 1× PBS, trypsinized for 5 min in 37 °C shaker, and spun down. Cells were then resuspended in 100 µL 1× PBS and placed on ice.

To place the DNA fibers, 2 μL of each sample was placed on a slide in a drop, and 7 μL of Spreading buffer was added on top and allowed to spread for 10 min. Cells were then slightly angled to allow the droplet to run vertically down the slide. This step should be very slow. The slides were allowed to dry for 20 min and then were fixed for 4 min in cold 3:1 methanol:glacial acetic acid solution. Slides were allowed to air dry for 5 min and were stored at 4 °C.

Slides were placed in a Coplin jar containing 2 N HCL for 30 min, covered from light. Slides were rinsed three times with 1× PBS and then placed in blocking buffer (5% bovine serum albumin (BSA), 0.1% TritonX in 1× PBS) for 1 h, covered from light. Slides were incubated for 1 h, covered from light, with two primary antibodies: rat monoclonal anti-BrdU (Abcam, Ab6326, recognizes CldU) and mouse anti-BrdU (BD, 347580, recognizes IdU) by placing the antibodies at a dilution of 1:100 in 5% BSA, adding 120 μL of this mixture dropwise to each slide, and carefully place a coverslip on top. Cover slips were removed, and slides were washed 3× with 1× PBS. Slides were then incubated, covered from light, with two secondary antibodies: goat anti-mouse IgG Alexa-Fluor-488 (green) and goat anti-mouse IgG Alexa-Fluor 597 (red) by placing antibodies at a dilution of 1:250 in 5% BSA, adding 120 μL of this mixture dropwise to the slide and placing a coverslip on top. Cover slips were removed and slides were rinsed 3× with PBS and allowed to air dry, covered from light. Slides were mounted using Prolong (Invitrogen, 36930).

Slides were imaged on a Nikon Ti2 Eclipse microscope using a 60× oil immersion objective. The length of red and green tracts on a single fiber was measured in Fiji software, and a ratio of red to green color tract length or total tract length was calculated.

### DNA Extraction.

Organoids were pelleted and then resuspended in 1× PBS. DNA was isolated using the Qiagen DNeasy Blood and Tissue Kit (Qiagen, 69504) according to the manufacturer’s protocol. Subcutaneous tumors were homogenized prior to DNA extraction.

### Subcutaneous Mouse Tumor Model.

NOD scid gamma mice were injected subcutaneously with 1 million cells per flank, and five mice were injected per organoid line, which included *Brca2^Δ/Δ^ Trp53^Δ/Δ^ Pten^Δ/Δ^* organoids expressing sgRNAs against *NT1, Cdt1 g2-14, Cdt1 g2-4,* and *Cdt1 g4-2*. Tumors were measured weekly in mm^3^, and when they reached endpoint size, they were harvested and flash frozen for future sample preparation.

### Sequencing of sgCdt1 Clones and Tumors.

Clones were isolated and expanded from sgCdt1 (g2 and g4) organoids using limiting dilution. These clones were injected subcutaneously into NOD scid gamma (Non-obese Diabetic, Severe Combined Immunodeficiency, and Gamma chain deficiency) mice as described above. Genomic DNA was extracted from organoids and tumors using the Qiagen DNeasy Blood and Tissue Kit (Qiagen, 69504) as described above. DNA samples were amplified using primers flanking the sgCdt1 cut sites and PCR products were isolated using the QIAquick Gel Extraction kit (Qiagen, 28704). Purified PCR products were sent to Integrated Genomics Operation (MSKCC) and CRISPResso2 analysis was performed to determine the editing outcomes from deep sequencing data (43). Allele fractions from pregraft organoid lines are reported (*SI Appendix*, Fig. S3) as well as from a subset of the subcutaneous tumors at the experimental endpoint (*SI Appendix*, Figs. S4–S6).

### Primary Antibodies

**Table:** 

Antibody	Cat. no	Dilution
β-actin	4907S, Cell Signaling Technology	1:10,000
Cas9	7A9-3A3, Cell Signaling Technology	1:1,000
Rb1	ab181616, Abcam	1:1,000
Pten	9559S, Cell Signaling Technology	1:1,000
p53	P53-CM5P-L, Leica	1:1,000
pCHK1 (S345)	2348S, Cell Signaling Technology	1:1,000
CHK1	2360S, Cell Signaling Technology	1:1,000
Cdc6	3387S, Cell Signaling Technology	1:1,000
Geminin	ab246509, Abcam	1:1,000
Cdt1	PA5-42861, Invitrogen	1:1,000
γH2AX	IF: ab1117, Abcam WB: 2,577, Cell Signaling Technology	WB 1:500
H2AX	2595S, Cell Signaling Technology	1:1,000

### Drug Table

**Table:** 

Drug name	Cat no.
Olaparib	LC Laboratories
AZD5305	HY-132167, MedChem Express
AF615	AOB16038, AOBIOUS
Dimethyl sulfoxide (DMSO, Vehicle)	BP231-100, Fisher Scientific

## Supplementary Material

Appendix 01 (PDF)

Dataset S01 (XLSX)

Dataset S02 (XLSX)

## Data Availability

All study data are included in the article and/or supporting information.
