# Supplementary material for: BRCA2 reversion mutation–independent resistance to PARP inhibition through impaired DNA prereplication complex function
Source: Proc Natl Acad Sci U S A. 2025 Jun 3;122(23):e2426743122. doi: 10.1073/pnas.2426743122 (PMC12167974; doi:10.1073/pnas.2426743122)
Supplement: Supplementary file 1 — Appendix 01 (PDF) [file pnas.2426743122.sapp.pdf]

PNAS

Supporting Information for

BRCA2 reversion mutation-independent resistance to PARP inhibition through impaired DNA pre-replication complex function

Kyrie Pappas[1], Matteo Ferrari[2], Perianne Smith[3], Subhiksha Nandakumar[4,5], Zahra Khan[1], Serina B. Young[6], Justin LaClair[7], Marco Vincenzo Russo[8], Emmet Huang-Hobbs[1], Nikolaus Schultz[1,4,5], Wassim Abida[9], Wouter Karthaus[10], Maria Jasin[2], Charles Sawyers[1,11]

Charles Sawyers, MD
Email: sawyersc@mskcc.org

This PDF file includes:

Figures S1 to S7
Legends for Spreadsheets S1 and S2
SI References

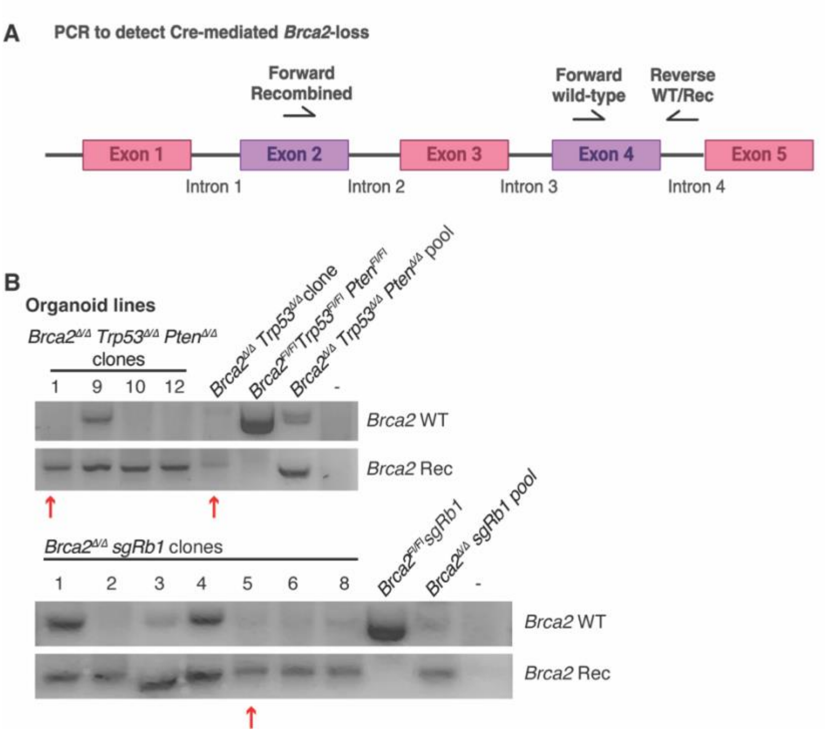

Figure S1: Brca2 genotyping of clones following *in vitro* Cre recombination in murine prostate organoid lines. (A) Brca2 knockout was confirmed in clones using PCR primers flanking the Lox sites on the *Brca2* gene (primer locations shown on gene diagram, created with BioRender.com). Amplification using the 'Forward Recombined' primer will only happen if recombination has occurred, and amplification using the 'Forward wild-type' primer will only occur if the wild-type allele is present. A knockout clone has a signal for the recombined PCR product and is negative for the wild-type PCR product. (B) Top: Brca2 genotyping of clones from *Brca2*$^{\Delta/\Delta}$ *Trp53*$^{\Delta/\Delta}$ *Pten*$^{\Delta/\Delta}$ and *Brca2*$^{\Delta/\Delta}$;*Trp53*$^{\Delta/\Delta}$ organoids. Bottom: Brca2 genotyping of clones from *Brca2*$^{\Delta/\Delta}$*sgRb1* organoids. Clones from each organoid line selected for studies are indicated by the red arrows.

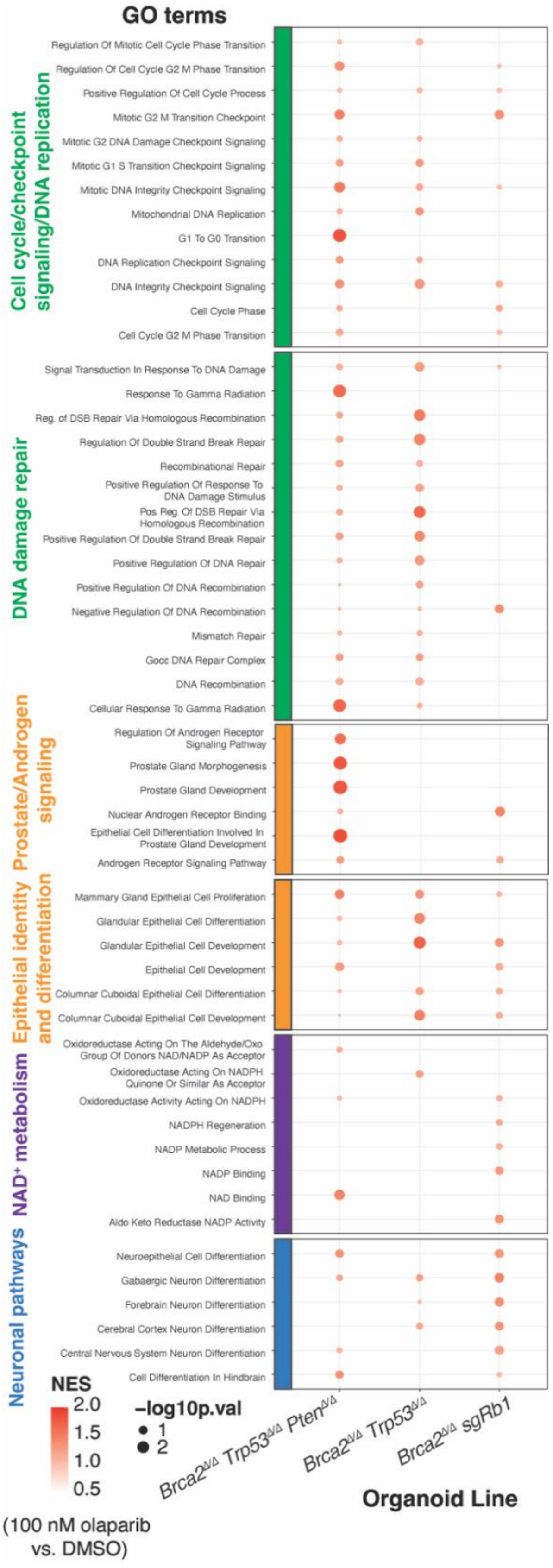

Figure S2: Gene set enrichment analysis (GSEA) of genome-wide Brie screen in Brca2-deficient murine prostate organoid lines. GSEA (1) was performed for all GO terms on the list of genes enriched in the genome-wide olaparib resistance screen above the 5% FDR threshold for 100 nM olaparib v. DMSO in *Brca2*$^{\Delta/\Delta}$ *sgRb1, Brca2*$^{\Delta/\Delta}$ *Trp53*$^{\Delta/\Delta}$ *Pten* $^{\Delta/\Delta}$*, and Brca2*$^{\Delta/\Delta}$ *Trp53*$^{\Delta/\Delta}$ murine prostate organoid lines. The red color gradient is the normalized enrichment score (NES), and the size of each circle represents the p-value. The scale for both parameters is indicated. Complete list of significant enrichments is reported in *SI Appendix*, Spreadsheet S2.

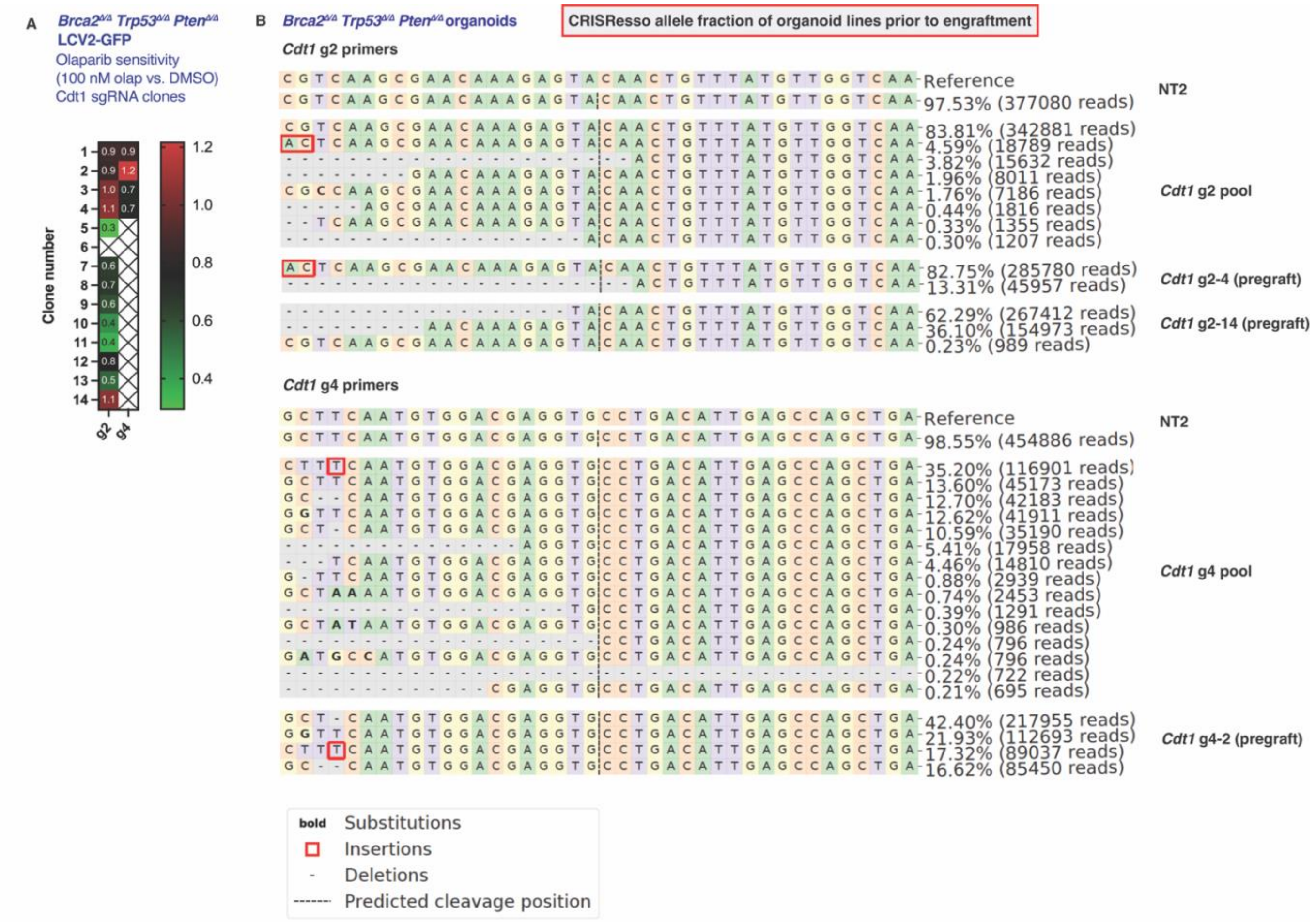

Figure S3: Characterization of *Cdt1* sgRNA clones in *Brca2*$^{\Delta/\Delta}$ *Trp53*$^{\Delta/\Delta}$ *Pten* $^{\Delta/\Delta/}$ organoids. Two sgRNAs against *Cdt1* (guides 2 and 4) were stably expressed in *Brca2*$^{\Delta/\Delta}$ *Trp53*$^{\Delta/\Delta}$ *Pten*$^{\Delta/\Delta}$ organoids, and single clones were isolated. (A)The olaparib sensitivity of clones was evaluated by treating with 100 nM olaparib or DMSO (vehicle) for 7 days, where viability was measured on the Incucyte. Values reported are the ratio of the olaparib/DMSO signals. Red clones are resistant and green clones are sensitive on heat map. Chosen clones (*Cdt1 g2-14, g2-4, g4*-2) had sensitivity values >1. (B) DNA was amplified around g2 or g4 cleavage sites using respective primers. CRISPResso2 (2) was used to analyze sequencing data around the *sgCdt1* cut sites for g2 and g4 in organoid cultures from both guide pools and clones. The allele sequences/fraction present for g2 and g4 pools and for each chosen clone (g2-14, g2-4, g4-2) are listed. Legend for deletions, insertions, and substitutions are reported, and the predicted cleavage site is indicated by the vertical dashed line.

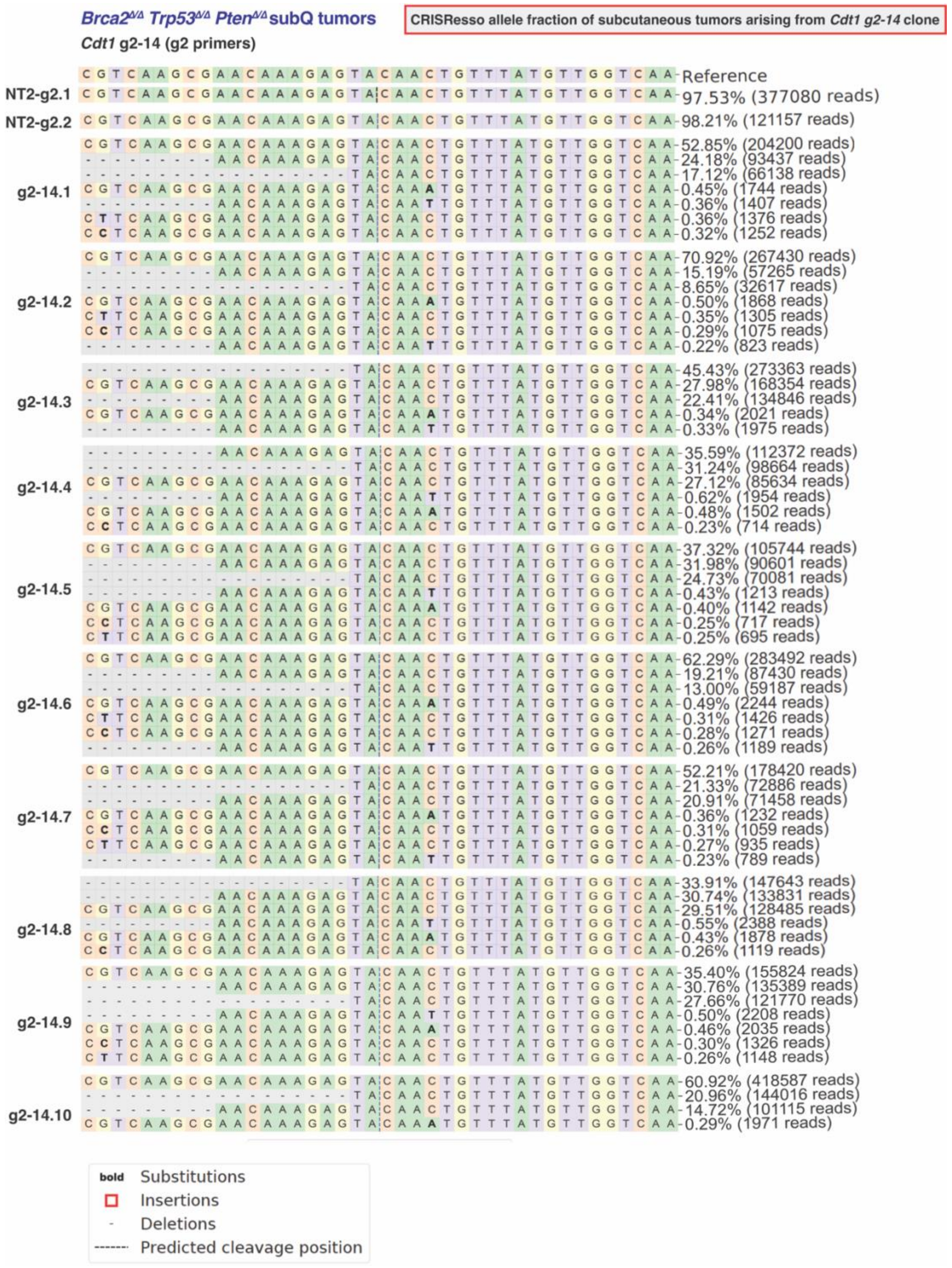

Figure S4: Sequence characterization of *Cdt1* g2-14 tumors in *Brca2*$^{\Delta/\Delta}$ *Trp53*$^{\Delta/\Delta}$ *Pten* $^{\Delta/\Delta/}$ organoids. DNA was amplified around g2 cleavage site. CRISPResso2 (2) was used to analyze sequencing data around the *sgCdt1* cut sites for *Cdt1 g2-14* subcutaneous tumors (n=10). The allele sequences/fraction

present are listed. Legend for deletions, insertions, and substitutions are reported, and the predicted cleavage site is indicated by the vertical dashed line.

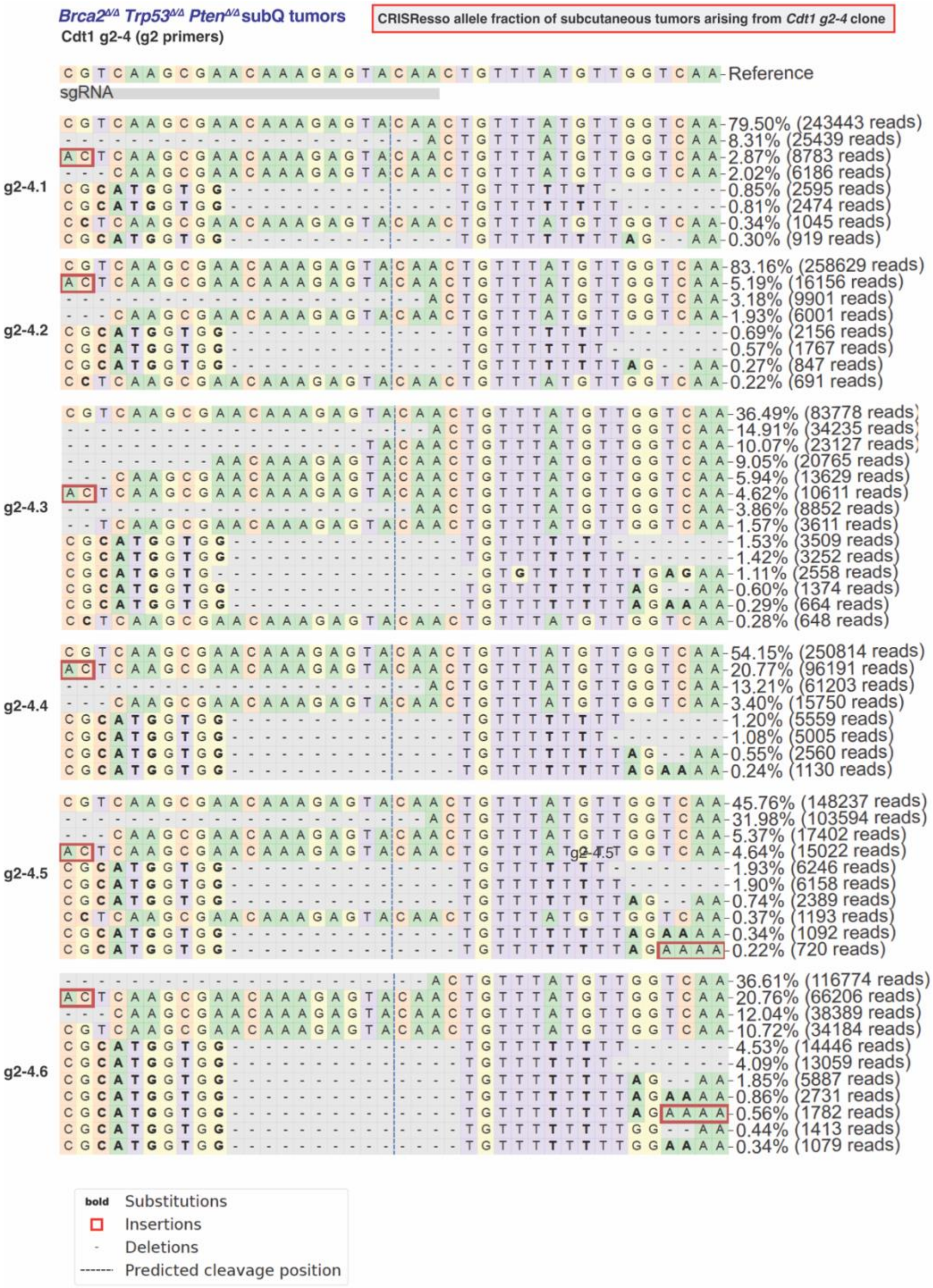

Figure S5: Sequence characterization of *Cdt1* g2-4 tumors in *Brca2*$^{\Delta/\Delta}$ *Trp53*$^{\Delta/\Delta}$ *Pten* $^{\Delta/\Delta}$ organoids. DNA was amplified around g2 cleavage site. CRISPResso2 (2) was used to analyze sequencing data around the *sgCdt1* cut sites for *Cdt1 g2-4* subcutaneous tumors (n=6). The allele sequences/fraction

present are listed. Legend for deletions, insertions, and substitutions are reported, and the predicted cleavage site is indicated by the vertical dashed line.

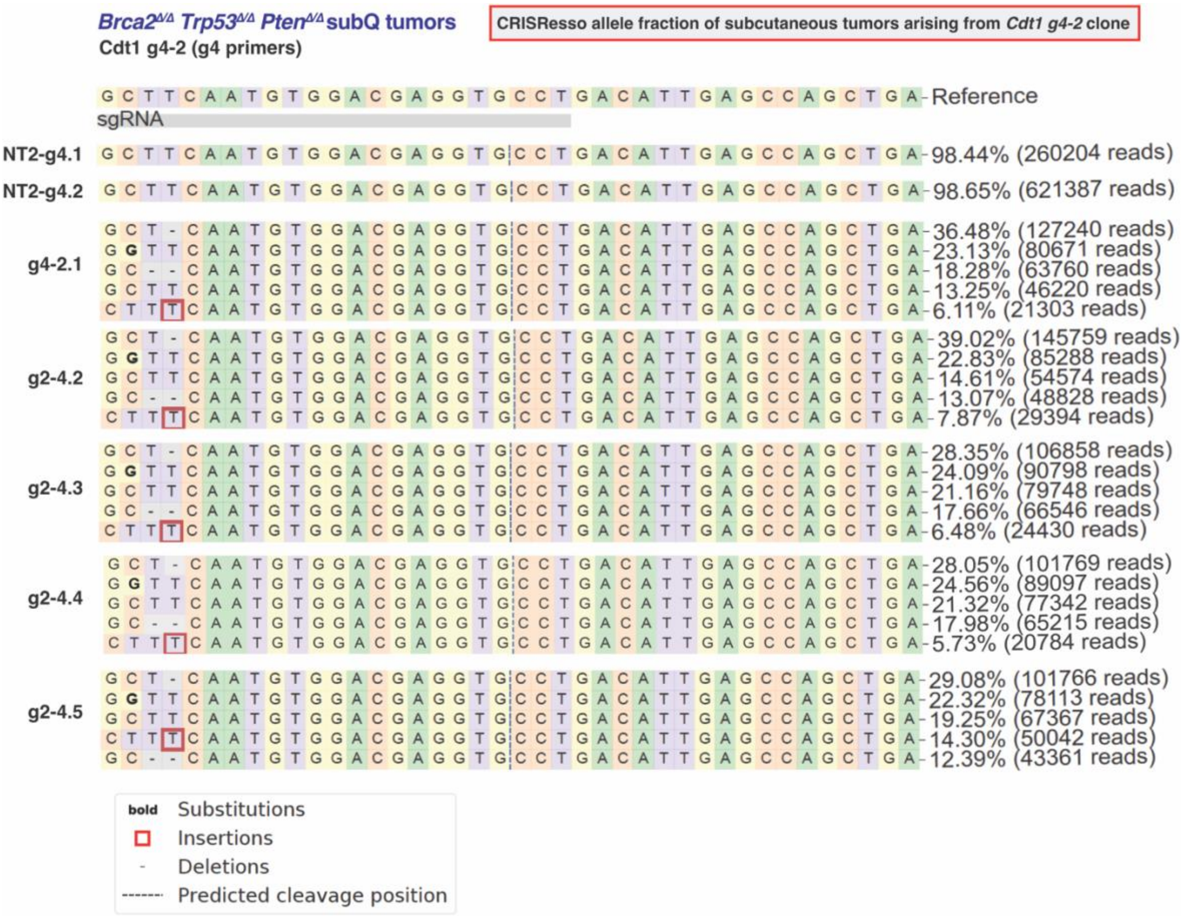

Figure S6: Sequence characterization of *Cdt1* g4-2 tumors in *Brca2*$^{\Delta/\Delta}$ *Trp53*$^{\Delta/\Delta}$ *Pten* $^{\Delta/\Delta/}$ organoids.

DNA was amplified around g4 cleavage site using respective primers. CRISPResso2 (2) was used to analyze sequencing data around the *sgCdt1* cut sites for *Cdt1 g4-2* subcutaneous tumors (n=5). The allele sequences/fraction present are listed. Legend for deletions, insertions, and substitutions are reported, and the predicted cleavage site is indicated by the vertical dashed line.

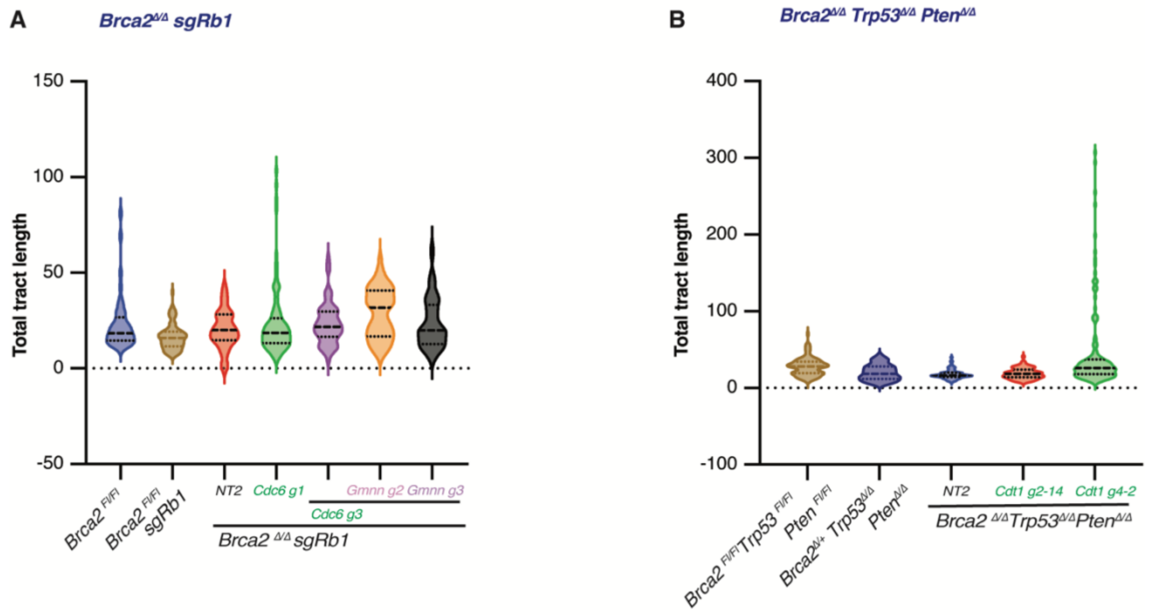

Figure S7: Tract length analysis of DNA fibers. DNA fiber analysis was performed where cells were treated with IdU (green) and CldU (red) for 20 minutes each, followed by hydroxyurea (HU) for 3h. Data is displayed as the total tract length (red + green), which is proportional to the speed of the replication fork. (A) DNA fiber tract length in *Brca2*$^{\Delta/\Delta}$ *sgRb1* organoids stably expressing the indicated sgRNAs *(sgNT1, sgNT2, Cdc6 g1, Cdc6 g3, Gmnn g2, Gmnn g3)* in LentiCRISPR-GFP. *Brca2*$^{Fl/Fl}$ *and Brca2*$^{Fl/Fl}$*sgRb1* organoids were included as controls. (B) DNA fiber tract length in *Brca2*$^{\Delta/\Delta}$ *Trp53*$^{\Delta/\Delta}$ *Pten*$^{\Delta/\Delta}$ organoids expressing the indicated sgRNAs (*sgNT1, Cdt1 g2-14, Cdt1 g4-2*) in LentiCRISPR-GFP. *Brca2*$^{Fl/Fl}$ *Trp53*$^{Fl/Fl}$ *Pten*$^{Fl/Fl,}$ and *Brca2*$^{\Delta/+}$ *Trp53*$^{\Delta/\Delta}$ *Pten*$^{\Delta/\Delta}$ organoids were included as controls.

Spreadsheet S1: Genome-wide Z-score analysis of enrichment/depletion from positive selection sgRNA screen for olaparib resistance performed in Brca2-deficient organoid series. *First tab:* Z-scores are reported for the following comparisons (100 nM olaparib vs. DMSO, 100 nM olaparib vs. T_0, and DMSO vs. T_0) in *Brca2*$^{\Delta/\Delta}$ *Trp53*$^{\Delta/\Delta}$ *Pten*$^{\Delta/\Delta}$, *Brca2*$^{\Delta/\Delta}$*Trp53*$^{\Delta/\Delta}$*, and Brca2*$^{\Delta/\Delta}$*sgRb1* organoids. *Second tab:* Significance cutoffs for enrichment/depletion Z-scores for the above comparisons based on a 5% FDR threshold. This analysis method was previously published and was performed by the Bioinformatics core at MSKCC (3).

Spreadsheet S2: Complete output from gene set enrichment analysis (GSEA) of Z-scores from genome-wide Brie screen in Brca2-deficient murine prostate organoid lines. GSEA (1) was performed for all GO terms on the ranked list of Z-scores from the genome-wide olaparib resistance screen above the 5% FDR threshold for 100 nM olaparib v. DMSO in *Brca2*$^{\Delta/\Delta}$ *sgRb1, Brca2*$^{\Delta/\Delta}$ *Trp53*$^{\Delta/\Delta}$ *Pten* $^{\Delta/\Delta}$*, and Brca2*$^{\Delta/\Delta}$ *Trp53*$^{\Delta/\Delta}$ murine prostate organoid lines. The name of each GO term, normalized enrichment score (NES), and the p-value that were used in Figure S2 (among other standard outputs) are reported.

References:

1. A. Subramanian *et al.*, Gene set enrichment analysis: a knowledge-based approach for interpreting genome-wide expression profiles. *Proc Natl Acad Sci U S A* **102**, 15545-15550 (2005).
2. L. Thorrez, H. Vandenburgh, CRISPResso2 provides accurate and rapid genome editing sequence analysis. *Nature biotechnology* **37**, 215-216 (2019).
3. R. Romero *et al.*, Keap1 mutation renders lung adenocarcinomas dependent on Slc33a1. *Nat Cancer* **1**, 589-602 (2020).
